# A Metallomic Approach to Assess Associations of Plasma Metal Levels with Amnestic Mild Cognitive Impairment and Alzheimer’s Disease: An Exploratory Study

**DOI:** 10.3390/jcm11133655

**Published:** 2022-06-24

**Authors:** Yu-Kai Lin, Chih-Sung Liang, Chia-Kuang Tsai, Chia-Lin Tsai, Jiunn-Tay Lee, Yueh-Feng Sung, Chung-Hsing Chou, Hung-Sheng Shang, Bing-Heng Yang, Guan-Yu Lin, Ming-Wei Su, Fu-Chi Yang

**Affiliations:** 1 Department of Neurology, Tri-Service General Hospital, National Defense Medical Center, Taipei 114, Taiwan; yukai0907@ndmctsgh.edu.tw (Y.-K.L.); jiakuang@mail.ndmctsgh.edu.tw (C.-K.T.); tsaicl0912@yahoo.com.tw (C.-L.T.); jiunntay@gmail.com (J.-T.L.); sungyf@mail.ndmctsgh.edu.tw (Y.-F.S.); choutpe@yahoo.com.tw (C.-H.C.); yuzi711@gmail.com (G.-Y.L.); 2 Graduate Institute of Medical Sciences, National Defense Medical Center, Taipei 114, Taiwan; lcsyfw@gmail.com; 3 Department of Psychiatry, Beitou Branch, Tri-Service General Hospital, National Defense Medical Center, Taipei 112, Taiwan; 4 Division of Clinical Pathology, Department of Pathology, Tri-Service General Hospital, National Defense Medical Center, Taipei 114, Taiwan; iamkeith001@gmail.com (H.-S.S.); rodancer@mail.ndmctsgh.edu.tw (B.-H.Y.); 5 Department of Neurology, Songshan Branch, Tri-Service General Hospital, National Defense Medical Center, Taipei 105, Taiwan; 6 Institute of Biomedical Sciences, Academia Sinica, Taipei 115, Taiwan; wei@ibms.sinica.edu.tw

**Keywords:** Alzheimer’s disease, mild cognitive impairment, trace metals, biomarkers, in vivo assessment

## Abstract

Alzheimer’s disease (AD) involves the abnormal activity of transition metals and metal ion dyshomeostasis; however, the potential of trace metal biomarkers in predicting cognitive decline has not been evaluated. This study aimed to assess the potential of 36 trace elements in predicting cognitive decline in patients with amnestic mild cognitive impairment (aMCI) or AD. Participants (9 controls, 23 aMCI due to AD, and 8 AD dementia) underwent comprehensive cognitive tests, including the Mini-Mental State Examination (MMSE) and trace metal analysis. The correlations between the plasma trace element levels and annual MMSE changes during follow-up were analyzed. We found that an increase in disease severity was linked to lower plasma levels of boron (B), bismuth (Bi), thorium (Th), and uranium (U) (adjusted *p* < 0.05). Higher baseline calcium levels (r = 0.50, *p* = 0.026) were associated with less annual cognitive decline; those of B (r = −0.70, *p* = 0.001), zirconium (r = −0.58, *p* = 0.007), and Th (r = −0.52, *p* = 0.020) with rapid annual cognitive decline in the aMCI group; and those of manganese (r = −0.91, *p* = 0.035) with rapid annual cognitive decline in the AD group. Overall, our exploratory study suggests that plasma metal levels have great potential as in vivo biomarkers for aMCI and AD. Larger sample studies are necessary to confirm these results.

## 1. Background

Alzheimer’s disease (AD) is a chronic progressive neurodegenerative disease that is usually mild at first and gradually worsens over time. It is the most common cause of dementia [1]. AD typically presents with amnestic syndrome, characterized by poor learning ability with memory loss or atypical variants, manifested as an early impairment of language function (aphasic variant), visuospatial function (posterior cortical atrophy), executive function (frontal/behavioral-comportamental variant), and motor function (corticobasal syndrome) [2].

Mild cognitive impairment (MCI) is an intermediate state between the cognitive changes observed in normal cognition and symptomatic pre-dementia [3]. Patients with MCI demonstrate unexpected cognitive impairments, based on their age and education level. However, in MCI, the disease severity does not meet the criteria for dementia [4]. A meta-analysis of 34 studies reported a 10–20% prevalence of MCI in adults aged ≥65 years [5]. Amnestic MCI (aMCI) is frequently observed as a prodromal stage of AD, with an annual conversion rate of up to 25% [4,6]. The aforementioned statistics highlight the importance of early diagnosis and intervention for patients with aMCI [7].

The National Institute on Aging and Alzheimer’s Association (NIA-AA) as well as the International Working Group have focused on a biomarker-based definition of AD, emphasizing on the importance of β-amyloid (Aβ) deposition, pathological tau, and neurodegeneration in the AD continuum [8,9]. The accepted biomarkers are amyloid positron emission tomography (PET) ligand binding, atrophy on structural magnetic resonance imaging (MRI), hypometabolism in fluorodeoxyglucose (FDG)–PET, and several cerebrospinal fluid (CSF) proteins, such as low levels of Aβ42 (or a Aβ_42_/Aβ_40_ ratio), elevated total tau (T-tau), and elevated phosphorylated tau (P-tau). Nevertheless, CSF collection is an invasive procedure and should only be performed by a technical physician, which limits access. Similarly, the high cost and labor-intensiveness of neuroimaging also limits the widespread application in primary care or clinical office-based settings. For this reason, there is limited evidence for the inclusion of CSF biomarkers or PET markers in MCI and AD diagnosis during routine clinical practice [10]. Therefore, less invasive and economical blood-based biomarker testing, as well as neuropsychological testing, genetic, clinical, and demographic information will likely play an important role in population screening in the future.

Abnormal transition metal activity plays a critical role in the pathogenesis of AD [11,12,13]. Dyshomeostasis and the concentration of metal ions in neurofibrillary tangles, senile plaques, and the CSF support this concept [14]. Specifically, dyshomeostasis and the generation of toxic Aβ oligomers are likely responsible for the AD-associated synaptic dysfunction. Therefore, the inhibition or prevention of amyloid plaque aggregation, the pathological hallmark of AD, may be treated through the targeting of metal ions, metal complexes, or metal-protein compounds, such as metal chelators is a potential therapeutic implication. This highlights the significant role of metals in the etiology of AD [15]. A previous cohort study conducted in Portugal showed that higher levels of selenium and nickel are associated with lesser cognitive decline in elderly patients [16]. However, to date, the potential of trace metal biomarkers in predicting cognitive decline in the AD continuum has not been evaluated. This study examined whether the concentration of metals in the plasma could be used to serve as a tool for patients in the AD continuum.

This study had two stages. In step 1, we administered baseline neuropsychological tests and collected the genetic information and plasma concentrations of 36 metals of participants (healthy controls, aMCI patients, and AD patients). In step 2, we performed the second Mini-Mental State Examination (MMSE) during the follow-up. We aimed to assess the potential of 36 trace elements in predicting cognitive decline in patients with aMCI or AD. In addition, we used plasma levels of the trace elements to define optimal cut-off values in order to differentiate healthy elderly controls (HCs) from those with aMCI or AD.

## 2. Methods

### 2.1. Patients

**Figure 1:**
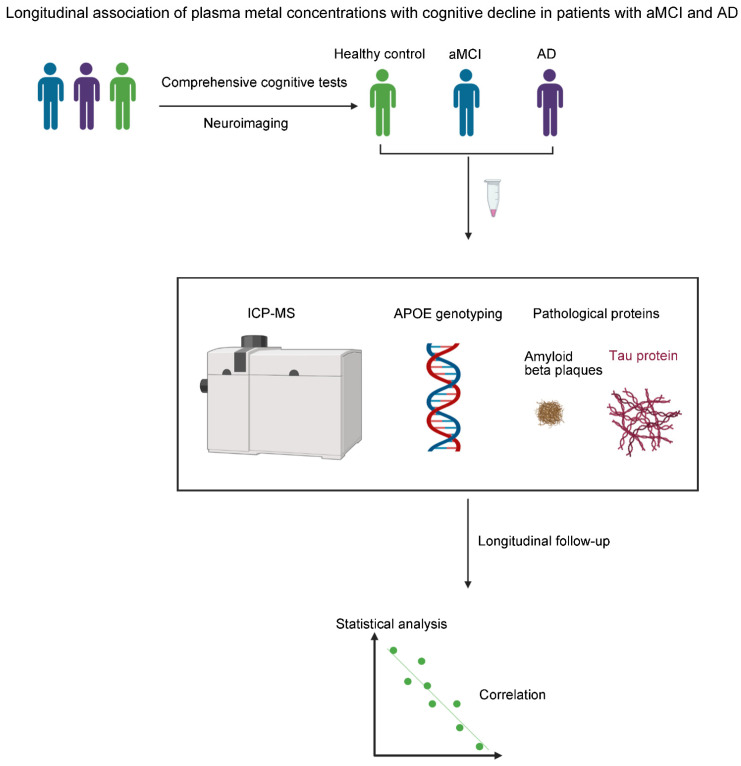
Schematic diagram of the study methodology and procedures. We recruited participants from the memory clinic at the Tri-Service General Hospital. After comprehensive cognitive tests and neuroimaging examination, the participants were divided into three groups (healthy control, aMCI, and AD). Blood samples were collected. ICP-MS, single nucleotide polymorphism (SNP) arrays, and immunomagnetic reduction (IMR) assays were used to evaluate plasma metal levels, APOE genotypes, and plasma pathological protein levels, respectively. In addition, statistical analyses were used to assess the correlation between metal levels and cognitive decline in patients with aMCI and AD. aMCI, amnestic mild cognitive impairment; AD, Alzheimer’s disease; ICP-MS, inductively coupled plasma-mass spectrometry; APOE, apolipoprotein E.

This observational cross-sectional study with longitudinal follow-up enrolled 40 subjects who attended the memory clinic at the Tri-Service General Hospital (TSGH) of the National Defense Medical Center, Taiwan between 1 January 2019 and 31 October 2020. The research methodology and procedures were summarized and shown in Figure 1. Of these subjects, there were 9 healthy controls (HCs), 23 patients with aMCI, and 8 with AD. The inclusion criteria were as follows: (1) age ≥ 60 years and (2) with negative results in physical and neurological examinations and laboratory tests (creatinine, fasting blood sugar, vitamin B12, folic acid, free-thyroxine 4 and high-sensitivity thyroid-stimulating hormone; serologic test for syphilis, white blood cell, red blood cell, hemoglobin, mean corpuscular volume, mean corpuscular hemoglobin, mean corpuscular hemoglobin concentration, and platelet count). We conducted brain imaging (brain computed tomography or MRI) to rule out non-Alzheimer’s disorders.

The exclusion criteria were as follows: (1) a history of major or uncontrolled medical diseases, such as heart failure, chronic obstructive pulmonary disease, liver cirrhosis, renal failure, sepsis, poorly controlled diabetes (hemoglobin A1c > 8.5), myocardial infarction, or malignancy; (2) substance abuse; (3) a history of major neurological diseases, such as stroke or Parkinson’s disease; (4) Geriatric Depression Scale score (short form) > 9 or modified Rankin Scale scores > 3; and (5) a history of major psychiatric illness that can impair cognitive function, such as major depressive disorder, bipolar disorder, or schizophrenia.

Following their written informed consent, the participants underwent assessment by the MMSE, Clinical Dementia Rating (CDR), short-form Geriatric Depression Scale (GDS-S), verbal fluency test (VFT), Hopkins Verbal Learning Test (HVLT), forward and backward digit span, Trail Making Test Part A (TMTA), Modified Boston Naming Test (MBNT), and Hachinski Ischemia Scale (HIS). The second MMSE was performed at a 1-year follow-up.

We classified the participants into the control, aMCI, and AD groups according to the results of HVLT, MMSE, and CDR, and the recommendations of the NIA-AA workgroups on diagnostic guidelines for AD and aMCI [17,18]. AD diagnosis was based on the following criteria: (1) NIA-AA criteria [18]; (2) CDR ≥ 0.5 [bc MMSE ≤ 26 (middle school), ≤22 (primary school), or ≤19 (illiteracy)); (3) HIS ≤ 3; and (4) HVLT ≤ 19 [19]. In contrast, aMCI diagnosis was based on the following criteria: (1) NIA-AA criteria [17]; (2) CDR = 0.5, memory item score of 0.5; (3) MMSE > 26 (middle school), >22 (primary school), or >19 (illiteracy); (4) HIS ≤ 3; and (5) HVLT ≤ 22 [19]. The healthy controls were required to meet the following criteria: (1) no active neurological or psychiatric disorders; (2) no psychotropic drugs; (3) MMSE > 26 (middle school), >22 (primary school), or >19 (illiteracy); and (4) CDR score = 0. All participants or their primary caregivers signed a written informed consent form following a complete written and verbal explanation of the study. The study protocol was approved by the Institutional Review Board of the TSGH.

### 2.2. Measuring the Plasma Trace Elements

We collected the venous blood in heparinized vacutainer BD tubes (Becton Dickinson Labware, Franklin Lakes, NJ, USA) and stored them at −20 °C until the time of analysis. We quantified the trace elements using an Agilent 7800 ICP-MS instrument (Agilent Technologies, Santa Clara, CA, USA).

Prior to the analysis, 100 μL blood plasma were diluted (1:50 *v*:*v*) with a diluent comprising 0.05% Triton X-100 (Sigma-Aldrich, St. Louis, MO, USA) and 1% HNO3 (ULTREX^®®^ ΙIUltrapure Reagent; J.T. Baker, Avantor, Radnor Township, PA, USA) in 18.2 MΩ cm distilled deionized water.

We calibrated the system using standard solutions with different concentrations of trace elements prepared from Certipur^®^ Certified Reference Material (Merck, Whitehouse Station, NJ, USA). We used metal solutions with final concentrations of 0.10, 20, 30, 40 and 50 μg/L for external calibration of the system. We performed a laboratory quality control via a permanent analysis of the certified reference material of blood plasma (ClinChek^®^ Plasma/Whole Blood Control for Trace Elements; RECIPE Chemicals + Instruments GmbH, Munich, Germany).

### 2.3. ApoE Genotyping

To efficiently obtain genetic information from samples collected from Taiwanese patients of Han Chinese ethnicity, the Taiwan Biobank (TWB) designed the TWB genotype array, based on the Affymetrix Axiom genotyping platform. The TWB genotype array enabled good-quality genotyping. Two single-nucleotide polymorphisms (SNPs, rs429358 and rs7412) defining Apo E isoforms were genotyped using the TWB array.

### 2.4. Plasma Biomarker Assays

Plasma amyloid β 1–40 (Aβ_1–40_), Aβ_1–42_, total Tau protein (t-Tau), phosphorylated Tau protein (Serine 181) (pTau) and total α-synuclein (α-Syn) were measured using immunomagnetic reduction (IMR). The assay procedures and protocol of IMR have been described in detail previously [20]. Every kind of biomarker in a plasma sample was assayed using IMR kits (MF-AB0-0060, MF-AB2-0060, MF-TAU-0060, MF-PT1-0060, MF-ASC-0060; MagQu, New Taipei City, Taiwan) and the IMR analyzer (XacPro-S, MagQu). Assays were done in duplicate for each biomarker of a sample. The mean value of the duplicated measurements was reported. For a single measurement, 80-/60-/80-/80-/80-μL reagent was mixed with 40-/60-/40-/40-/40-μL plasma for assaying Aβ_1–40_/Aβ_1–42_/t-Tau/pTau/α-Syn.

### 2.5. Statistical Analyses

We compared the demographics, scores of cognitive tests, and IMR data between the binary study groups (i.e., control vs. patients; aMCI vs. AD) using the independent-samples *t*-test and Fisher’s exact test for continuous variables (age, education, body mass index, cognitive tests, and IMR data) and the categorical variable (sex), respectively. Considering the lack of normality, levels of trace metals among the study groups were compared using the non-parametric Kruskal–Wallis test. Multiple comparisons (post hoc) between two groups (aMCI vs. control; AD vs. control) were performed when the overall test was statistically significant. Moreover, we evaluated the linear trend of trace metals across the disease groups using the Jonckheere–Terpstra test. In addition, we further adjusted for age when evaluating the group difference (pairwise comparison) and the trend analysis in the cognitive tests and trace metals, as age significantly differed between groups.

We also evaluated the utility of individual trace metals in differentiating between the disease groups (aMCI vs. control; AD vs. control; and AD vs. aMCI) using the area under the receiver operating characteristic (ROC) curve (AUC). The 95% confidence interval of AUC was calculated using DeLong’s method. We used the Youden index to determine the optimal cut-off value.

The trace metals with significant differentiation ability in the previous ROC analyses were selected for further analyses. The association between those trace metals and the annual change of the MMSE score ([second assessment − first assessment]/follow-up year) in either disease group (aMCI or AD) was further assessed using a partial correlation with an adjustment for the age, education level, and body mass index. All tests were two-tailed and a *p*-value < 0.05 was considered statistically significant. Data analyses were conducted using SPSS 25 (IBM, Armonk, NY, USA).

## 3. Results

### 3.1. Patient Profiles

Of the total 40 subjects, 9 were non-disease controls. The remaining were comprised of 23 and 8 patients with aMCI and AD, respectively. There was no difference in the sex distribution between groups (control vs. patients; aMCI vs. AD). Subjects in the patient group were older than those in the control group (79.5 ± 8.1 vs. 67.0 ± 6.3 years), whereas the age was comparable between the aMCI and AD groups. The patient group demonstrated poor scores on all cognitive tests (except the discrimination index) than the control group. In contrast, the AD group exhibited poorer scores than the aMCI group on several cognitive tests, including baseline MMSE, CDR sum of box score, discrimination index, and VFT. There was no significant difference in the *APOE* allele status and IMR data between the study groups. However, the aMCI and AD patients had higher trends in peripheral levels of t-Tau and Aβ1-42 × t-Tau than controls (Table 1).

### 3.2. Trace Elements

**Figure 2:**
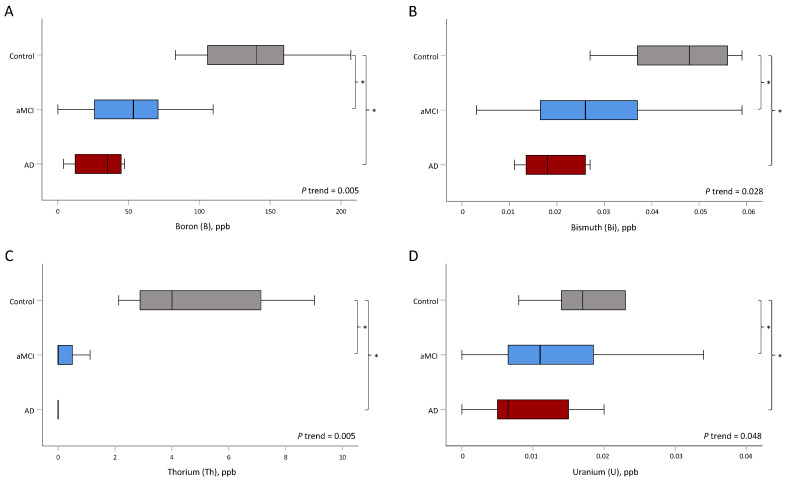
The distribution of trace metals was significantly different between aMCI and control groups and between AD and control groups. Asterisks (*) indicate statistical significance (*p* < 0.05). (**A**) Boron, (**B**) bismuth, (**C**) thorium, and (**D**) uranium. aMCI, amnestic mild cognitive impairment; AD, Alzheimer’s disease.

**Figure 3:**
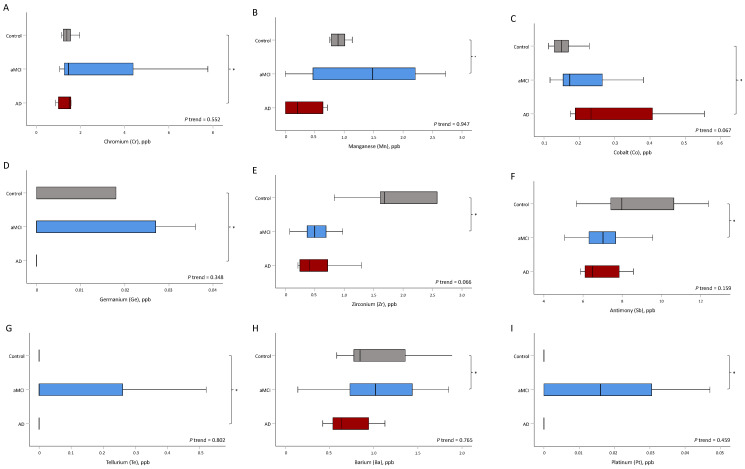
The distribution of trace metals was significantly different between aMCI and control groups and between AD and control groups. Asterisks (*) indicate statistical significance (*p* < 0.05). (**A**) Chromium, (**B**) manganese, (**C**) cobalt, (**D**) germanium, (**E**) zirconium, (**F**) antimony, (**G**) tellurium, (**H**) barium, and (**I**) platinum. aMCI, amnestic mild cognitive impairment; AD, Alzheimer’s disease.

An increase in the disease severity was associated with lowered B, Bi, Th, and U levels (all of adjusted *p* for trend < 0.05) (Table 2 and Figure 2). The levels of B (*p* = 0.009), Bi (*p* = 0.034), Th (*p* = 0.002), and U (*p* = 0.034) were significantly different between the aMCI and control groups, as well as between the AD and control groups (B, *p* = 0.012; Bi, *p* = 0.007; Th, *p* = 0.043; and U, *p* = 0.005). However, the levels of Mn (*p* = 0.028), Zr (*p* = 0.034), Sb (*p* = 0.038), Ba (*p* = 0.022), and Pt (*p* = 0.038) were only significantly different between the aMCI and control groups. In contrast, the levels of Cr (*p* = 0.043), Co (*p* = 0.027), Ge (*p* = 0.002), and Te (*p* = 0.027) were only significantly different between the AD and control groups (Table 2 and Figure 3).

### 3.3. The Utility of Trace Metals to Differentiate between the Disease Groups

We evaluated the utility of trace metals in differentiating between the disease groups (aMCI vs. control; AD vs. control; and AD vs. aMCI). The levels of B, Hg, and Th could differentiate between all disease groups, including between aMCI and AD. In contrast, the levels of Ca, Zr, W, Tl, Bi, and U could differentiate both aMCI and AD from the control group. In addition, the levels of B, Mn, Co, Cu, Ge, Se, Ba, Pt, Hg, Pb, and Th could distinguish between the aMCI and AD groups (Supplementary Table S1). The optimal cut-off value of the selected trace metals with satisfying differentiation ability (AUC > 70.0%) and the corresponding sensitivity/specificity are displayed in Supplementary Table S2.

### 3.4. The Association between Trace Metals and an Annual Change in MMSE Scores

We eventually selected the trace metals with significant differentiating ability to evaluate their association with the annual change in MMSE scores. Higher levels of B (r = −0.70, *p* = 0.001), Zr (r = −0.58, *p* = 0.007), and Th (r = −0.52, *p* = 0.020) were significantly associated with a greater cognitive decline in the aMCI group. In contrast, higher levels of Ca (r = 0.50, *p* = 0.026) were significantly associated with a less cognitive decline in the aforementioned group. Higher levels of Mn (r = −0.91, *p* = 0.035) were associated with a greater cognitive decline in the AD group (Table 3).

## 4. Discussion

This is the first clinical study to evaluate the relationship between multiple plasma metal levels and cognitive decline in healthy participants as well as those with aMCI or AD. The plasma concentrations of B, Bi, Th, and U decreased with an increase in the disease severity. Moreover, B, Bi, Th, and U levels were significantly different between patients with aMCI as well as AD and the healthy controls. The ROC analyses revealed that the plasma concentrations of B, Hg, and Th could differentiate between the disease groups (aMCI vs. control; AD vs. control; and AD vs. aMCI). B demonstrated high AUCs for aMCI versus the controls (97.6%, cut-off value: ≤73.1 μg/L) and AD versus the controls (100%, cut-off value: ≤47.1 μg/L). Hg revealed the highest AUC to differentiate AD from aMCI (79.9%, cut-off value: ≤1.02 μg/L). Following an adjustment for the potential confounding factors (age, education level and body mass index) in the aMCI group, while higher baseline levels of Ca were associated with a smaller cognitive decline, those of B, Zr, and Th were associated with a rapid cognitive decline. In contrast, higher baseline levels of Mn were associated with a rapid cognitive decline in the AD group.

B levels were negatively associated with aMCI and AD. B is an essential trace element, abundant in fruits, vegetables, walnuts, and pulses. Recent animal and human studies have reported that long-term dietary supplementation with walnuts may reduce the risk or delay the progression of aMCI and AD [21,22]. There is increasing evidence for the beneficial effects of B on human health, particularly in promoting hormone and immune response, inflammation, oxidative stress regulation, and central nervous system function [23]. Furthermore, B deprivation leads to poor performance in tasks, such as movement speed and flexibility, attention, and short-term memory in older adults [24]. In other words, the aforementioned studies highlight an association between B levels and cognitive function. In addition, B plays an important role in human brain function and cognitive protection. Conversely, our study found that higher plasma B levels were associated with greater cognitive decline in the aMCI group. However, our study had a modest sample size with a one-year follow-up. Further large-scale studies with longer follow-up are warranted to establish a potential relationship between plasma B levels and cognitive decline.

Plasma Th levels were negatively associated with aMCI and AD. Moreover, higher baseline levels of Th were associated with a faster cognitive decline. An animal study reported that Th-treated mice demonstrated impaired learning and memory performance, similar to our results [25]. Furthermore, it resulted in the activation of acetylcholinesterase in the mouse brain [25]. This necessitates further research on humans to reveal the underlying association between Th and cognitive function.

Plasma U concentrations were negatively associated with aMCI and AD. Daily dietary intake as well as water consumption are the most common ways of ingesting U. Root crops, such as potatoes and sweet potatoes, contribute the highest U content in the diet [26]. Moreover, sweet potato anthocyanins can enhance memory and improve cognitive deficits, which in turn may be related to its antioxidant properties [27,28].

Ca is an essential element needed for normal brain function, but Ca dysregulation plays an important role in AD pathology [29,30]. Regarding the protective effect of Ca on cognitive function, our study found that higher baseline levels of Ca were associated with less cognitive decline in patients with aMCI. Ca is an essential element and Ca signaling regulates neuronal metabolism and energy production which is necessary to maintain synaptic transmission [30]. In addition, Ca signaling in neurons is essential for neurotransmission and producing long-term potentiation which forms the biological basis of memory and learning through the gradual enhancement of synapses [31]. Regarding the negative effects of Ca on AD pathology, our study found that, according to an unadjusted trend analysis, Ca levels were lowest in the healthy control group and highest in the aMCI and AD groups. Similarly, a longitudinal population-based study from Sweden revealed that women receiving Ca supplements are at higher risk of developing dementia (odds ratio, 2.10; *p* = 0.046) [32]. Recent studies have reported on the association between the disruption of intracellular Ca^2+^ homeostasis and the neuropathology of AD, memory loss, and cognitive dysfunction [29,33]. Increased intracellular Ca in the endoplasmic reticulum (ER) is a possible mechanism by which presenilin mutations disrupt intracellular Ca signaling. Furthermore, preclinical studies have revealed that excess ER Ca^2+^ release through the inositol 1,4,5-trisphosphate receptor or the ryanodine receptor is related to tau and amyloid pathology, and contributes to memory and learning deficits [34,35]. Therefore, Ca dyshomeostasis plays a critical role in the pathogenesis of AD.

We observed an inverse association between Hg levels and both aMCI and AD. There are three major groups of Hg compounds, namely elemental, inorganic, and organic. Hg is converted to methylmercury by bacteria, which enters the food chain and bioaccumulates in predatory fish [36]. Fish consumption is the primary source of methylmercury exposure [36]. Seafood, including shellfish and finfish, is the largest contributor to organic Hg exposure in the human population. A systematic review mentioned that long-chain omega-3 fatty acids in a high-fish diet can delay cognitive decline in elderly individuals, without dementia [37]. The serum Hg levels of our subjects were within the normal range, thus indicating a normal dietary intake (normal value: <20 μg/L for women aged ≥ 50 years and men aged > 18 years) [38]. Nonetheless, ICP-MS can only detect total Hg and fails to distinguish between the organic and inorganic forms. However, compared with healthy controls, patients with aMCI or AD may likely reduce their seafood intake, thereby reducing organic methylmercury exposure [39]. We did not use a detailed food frequency questionnaire, including the types, frequency, and amount of seafood intake. This made it difficult to explain the inverse correlation between Hg levels and aMCI and AD, thus necessitating further investigation.

Our study established an association between higher baseline levels of Mn and rapid annual cognitive decline in patients with AD. Mn is an essential metal that maintains the normal functions of the human body. However, increased Mn levels in the brain are associated with impaired motor coordination, memory deficits, psychiatric disorders, and Parkinson’s disease [40,41,42]. An animal study reported that the overexpression of Aβ in transgenic mice led to Mn accumulation in the brain, thus suggesting a role of Aβ in Mn homeostasis and neurotoxicity [43]. A study conducted in China further mentioned that people with higher plasma Mn concentrations were associated with higher plasma Aβ peptides levels [44]. The aforementioned studies suggest a relationship between Mn and AD, and the presence of shared pathophysiological mechanisms.

Li was used as a drug for treatment of bipolar disorder. A prior systematic review showed that Li may have neuroprotective and neurotoxic effects [45]. Regarding the mechanism of impact of Li on neuroprotection, Li may increase the choline and glycine levels of red blood cells in patients with AD [46]. A recent retrospective cohort study found an association between Li use and a reduced risk of developing dementia in people over the age of 50 [47]. A randomized clinical trial conducted in Brazil showed that long-term Li treatment at subtherapeutic concentrations (0.25–0.5 mEq/L) attenuates cognitive and functional decline in subjects with amnestic MCI. Furthermore, Li modifies CSF biomarkers of AD [48]. Regarding the mechanism of impact of Li on neurotoxic effect, Smith et al. reported a middle-aged man who presented with encephalopathy with tremor and myoclonic jerks secondary to Li intoxication [49]. Our study showed there was no significant difference in plasma Li levels between control and patient groups. More basic research data as well as clinical data concerning Li therapy is needed to clarify these inconsistent results.

Interestingly, recent preclinical animal studies have shown that some trace elements can attenuate cognitive deficits in animal models of AD [50,51]. Akhtar et al. [50] revealed attenuation of cognitive deficit in intracerebroventricular injection of streptozotocin (ICZ-STZ) rats treated with chromium picolinate. In addition, chromium picolinate reversed AD pathology by improving memory, reducing oxidative stress, mitochondrial dysfunction, neuroinflammation, and upregulating insulin signaling [50]. In addition, sodium orthovanadate improved learning and memory ability in an ICZ-STZ rat model of AD by the upregulation of the IRS-1/PI3K/AKT/GSK-3β pathway [51].

The strengths of our study include the robust statistical analysis, detailed cognitive examinations, prospective design, and large response rate at follow-up. However, our study had some limitations, which should be considered while interpreting the findings. First, the participants were recruited in a tertiary medical center. Therefore, our results may not be generalized to other populations, such as elderly people living in the community. However, there was also some strength of the single-center design, which made it possible to systematically and uniformly collect all the data of all participants. In addition, the cognitive assessment conducted by the board-certified clinical psychologist, making information bias less likely. Second, there was no significant difference in the discrimination index between the control and patient groups (although the control group had higher trends in discrimination index than the patient group), but the AD group exhibited a lower discrimination index than the aMCI group. This difference may be due to the small sample size. Further studies with larger sample sizes are needed to clarify this inconsistency. Third, we only measured the metals at a single timepoint, which may reflect a short period of exposure. It is uncertain whether the observed metal levels reflect exposure prior to the onset of cognitive impairment, or whether these levels are affected by the presence of aMCI or AD. Therefore, long-term serial measurements of trace metals may help researchers explore their relationship with cognitive decline.

## 5. Conclusions

This was the first exploratory study to compare the differentiating ability of trace elements biomarkers in patients with aMCI and AD. Several trace elements were significantly associated with the above-mentioned cognitive tests and annual cognitive changes in the patients. The plasma concentrations of B, Hg, and Th could satisfactorily detect different stages of cognitive function in healthy controls and patients with aMCI and AD. Moreover, higher baseline levels of B, Zr, and Th and Mn were associated with a rapid cognitive decline in patients with aMCI and AD, respectively. Further large-scale longitudinal studies are required to replicate our preliminary findings.

## Figures and Tables

**Table 1:** Baseline characteristics of the 40 enrolled participants according to disease group.

	Control	Patients		aMCI	AD	
Variable			*p*-Value			*p*-Value
**Demographics**						
Male	3 (33.3)	6 (19.4)	0.394	4 (17.4)	2 (25.0)	0.634
Age, years	67.0 ± 6.3	79.5 ± 8.1	<0.001	78.3 ± 7.8	82.9 ± 8.6	0.178
Education, years	10.9 ± 3.8	7.8 ± 4.8	0.090	7.3 ± 4.7	9.5 ± 5.1	0.266
Body mass index, kg/m^2^	23.4 ± 2.4	24.8 ± 3.9	0.308	25.2 ± 4.0	23.8 ± 3.9	0.400
**Cognitive tests**						
Baseline MMSE	29.3 ± 0.5	22.8 ± 5.3	0.001	23.9 ± 3.7	19.5 ± 7.7	0.039
CDR sum of box score	0.4 ± 0.3	2.6 ± 2.6	0.019	1.7 ± 1.1	5.1 ± 4.0	0.001
Hopkins Verbal Learning Test	22.0 ± 5.2	15.2 ± 5.4	0.002	16.0 ± 4.9	13.0 ± 6.7	0.184
Discrimination Index	11.1 ± 0.8	9.3 ± 2.9	0.073	9.9 ± 1.8	7.5 ± 4.6	0.041
Forward digit span	11.2 ± 1.6	8.4 ± 2.8	0.007	8.3 ± 2.8	8.6 ± 3.0	0.813
Backward digit span	6.9 ± 3.0	3.9 ± 2.8	0.008	4.1 ± 3.0	3.1 ± 2.4	0.395
Verbal fluency test	14.3 ± 2.2	9.7 ± 4.5	0.005	11.0 ± 4.2	6.1 ± 3.2	0.006
Modified Boston Naming Test	14.3 ± 0.9	12.9 ± 1.8	0.028	13.1 ± 1.5	12.4 ± 2.3	0.306
Trail Making Test Part A	53.9 ± 27.7	147.5 ± 101.9	0.010	136.2 ± 98.8	179.9 ± 110.4	0.304
**Apolipoprotein E** **ε** **2:** **ε** **3:** **ε** **4**	2:15:1 (11%:83%:6%)	3:48:11 (5%:77%:18%)	0.313	2:37:7(4%:81%:15%)	1:11:4 (6%:69%:25%)	0.625
**IMR data**						
t-Tau, pg/mL	23.5 ± 1.8	25.5 ± 3.6	0.125	25.5 ± 3.9	25.3 ± 2.8	0.891
Aβ_1–42_, pg/mL	16.9 ± 0.4	17.2 ± 0.8	0.360	17.1 ± 0.9	17.2 ± 0.7	0.964
p-Tau181, pg/mL	3.6 ± 0.4	3.8 ± 0.6	0.257	4.0 ± 0.5	3.5 ± 0.7	0.093
Aβ_1–40_, pg/mL	52.6 ± 4.9	52.3 ± 4.1	0.873	52.8 ± 4.2	50.9 ± 3.8	0.287
α-synuclein, fg/mL	108.5 ± 83.4	120.6 ± 65.6	0.648	124.7 ± 70.2	109.0 ± 52.4	0.569
Aβ_1–42_ × t-Tau	1.39 ± 0.10	1.48 ± 0.15	0.104	1.48 ± 0.16	1.47 ± 0.11	0.867
Aβ_1–42_ × Aβ_1-40_	0.32 ± 0.03	0.33 ± 0.03	0.592	0.33 ± 0.03	0.34 ± 0.03	0.370
p-Tau × t-Tau	0.15 ± 0.02	0.15 ± 0.02	0.807	0.16 ± 0.02	0.14 ± 0.02	0.068

Data are expressed as mean ± standard deviation or frequency (percentage). Abbreviations: *aMCI*, amnestic mild cognitive impairment due to AD; *AD*, Alzheimer’s disease; *MMSE*, Mini-Mental Status Examination; *CDR*, Clinical Dementia Rating; *IMR*, ultra-sensitive immunomagnetic reduction; *t-Tau*, total Tau; *Aβ*, amyloid β; *p-Tau181*, tau phosphorylated at threonine 181; *p-Tau, phosphorylated tau*.

**Table 2:** Trace metals of the enrolled participants according to disease group ^a^.

	Control	aMCI	AD	*p*-Value #		
Variable				*P* Trend	aMCI vs. Control	AD vs. Control
Li, μg/L	1.07 (0.73, 1.11)	1.21 (1.01, 1.70)	1.35 (0.73, 1.61)	0.989	0.414	0.847
Be, μg/L	0.92 (0.50, 0.98)	0.61 (0.51, 1.49)	0.64 (0.40, 1.15)	0.474	0.722	0.564
B, μg/L	141 (106, 160)	53 (24, 71)	35 (12, 45)	**0.005**	**0.009**	**0.012**
Al, μg/L	18.0 (15.0, 19.6)	14.5 (11.1, 17.9)	14.5 (13.2, 19.7)	0.299	0.950	0.248
Ca, mg/L	86.5 (84.6, 88.5)	93.1 (88.2, 99.1)	93.1 (89.0, 97.1)	0.291	0.173	0.441
V, μg/L	0.29 (0.18, 0.33)	0.21 (0.13, 0.26)	0.22 (0.17, 0.26)	0.477	0.201	0.564
Cr, μg/L	1.4 (1.2, 1.5)	1.5 (1.2, 4.4)	1.5 (1.0, 1.6)	0.552	0.414	**0.043**
Mn, μg/L	0.89 (0.78, 1.01)	1.48 (0.46, 2.26)	0.21 (0.00, 0.64)	0.947	**0.028**	0.773
Fe, μg/L	987 (904, 1140)	1360 (952, 1654)	1316 (987, 1708)	0.468	0.201	0.386
Co, μg/L	0.15 (0.13, 0.17)	0.17 (0.15, 0.27)	0.23 (0.19, 0.41)	0.067	0.267	**0.027**
Ni, μg/L	0.66 (0.45, 0.99)	0.91 (0.69, 1.56)	0.63 (0.45, 1.09)	0.821	0.107	1.000
Cu, μg/L	832 (693, 915)	956 (850, 1090)	788 (743, 977)	0.625	0.090	0.564
Zn, μg/L	780 (633, 842)	751 (679, 802)	740 (690, 830)	0.706	0.883	0.630
Ga, μg/L	0.071 (0.047, 0.095)	0.059 (0.024, 0.071)	0.036 (0.035, 0.036)	0.102	0.173	0.248
Ge, μg/L	0.00 (0.00, 0.02)	0.00 (0.00, 0.04)	0.00 (0.00, 0.00)	0.348	0.216	**0.002**
As, μg/L	4.9 (4.3, 9.4)	5.7 (4.7, 10.3)	5.5 (4.6, 8.8)	0.541	0.600	0.700
Se, μg/L	171 (158, 183)	175 (158, 191)	148 (141, 167)	0.368	0.414	0.124
Rb, μg/L	216 (209, 250)	219 (193, 243)	206 (162, 252)	0.599	0.917	0.700
Sr, μg/L	30.0 (26.9, 35.1)	34.9 (27.9, 43.9)	35.6 (32.4, 39.4)	0.265	0.304	0.102
Zr, μg/L	1.68 (1.61, 2.58)	0.50 (0.37, 0.70)	0.41 (0.25, 0.72)	0.066	**0.034**	0.248
Mo, μg/L	2.0 (1.0, 2.0)	2.1 (1.3, 2.8)	1.7 (1.2, 2.9)	0.932	0.753	0.441
Ag, μg/L	0.02 (0.00, 0.29)	0.03 (0.00, 0.13)	0.07 (0.00, 0.21)	0.514	0.867	0.700
Cd, μg/L	0.07 (0.04, 0.07)	0.07 (0.05, 0.14)	0.06 (0.04, 0.07)	0.499	0.630	0.386
Sn, μg/L	0.00 (0.00, 0.59)	0.00 (0.00, 0.12)	0.00 (0.00, 0.00)	0.978	0.216	0.500
Sb, μg/L	8.0 (7.4, 10.6)	7.0 (6.1, 7.7)	6.5 (6.1, 7.8)	0.159	**0.038**	0.211
Te, μg/L	0.00 (0.00, 0.00)	0.00 (0.00, 0.26)	0.00 (0.00, 0.00)	0.802	0.232	**0.027**
Ba, μg/L	0.8 (0.8, 1.4)	1.0 (0.7, 1.4)	0.6 (0.5, 0.9)	0.765	**0.022**	0.211
W, μg/L	95.4 (33.0, 230.3)	0.0 (0.0, 0.0)	0.0 (0.0, 0.0)	0.071	0.173	0.563
Pt, μg/L	0.00 (0.00, 0.00)	0.02 (0.00, 0.03)	0.00 (0.00, 0.00)	0.459	**0.038**	0.149
Au, μg/L	4.2 (1.9, 35.6)	0.0 (0.0, 0.0)	0.0 (0.0, 0.0)	0.211	0.516	0.290
Hg, μg/L	3.2 (2.2, 7.0)	1.8 (1.2, 2.2)	0.8 (0.6, 1.2)	0.102	0.850	0.700
Tl, μg/L	0.052 (0.033, 0.072)	0.028 (0.018, 0.044)	0.014 (0.010, 0.037)	0.073	0.126	0.124
Pb, μg/L	0.18 (0.17, 0.42)	1.47 (0.23, 1.97)	0.43 (0.00, 1.14)	0.652	0.209	0.441
Bi, μg/L	0.05 (0.04, 0.06)	0.03 (0.02, 0.04)	0.02 (0.01, 0.03)	**0.028**	**0.034**	**0.007**
Th, μg/L	4.0 (2.9, 7.1)	0.0 (0.0, 0.5)	0.0 (0.0, 0.0)	**0.005**	**0.002**	**0.043**
U, μg/L	0.017 (0.014, 0.023)	0.011 (0.006, 0.021)	0.007 (0.005, 0.015)	**0.048**	**0.034**	**0.005**

Abbreviations: aMCI, amnestic mild cognitive impairment; AD, Alzheimer’s disease; NA, not applicable; ^a^ Data are expressed as median [1st quartile, 3rd quartile]. # The analysis was adjusted for age.

**Table 3:** Association between trace metals and annual change of MMSE *.

Trace Metal	aMCI		AD	
	Partial Correlation #	*p*-Value	Partial Correlation #	*p*-Value
B	**−0.70**	**0.001**	−0.03	0.967
Al	−0.09	0.707	−0.01	0.982
Ca	**0.50**	**0.026**	−0.82	0.092
Mn	−0.35	0.133	**−0.91**	**0.035**
Co	−0.25	0.296	−0.37	0.545
Cu	−0.11	0.646	−0.44	0.454
Ga	0.10	0.676	−0.04	0.948
Ge	−0.03	0.889	NA	NA
Se	0.20	0.405	0.35	0.560
Zr	**−0.58**	**0.007**	−0.50	0.389
Sb	0.13	0.594	0.71	0.178
Ba	−0.25	0.298	−0.45	0.449
W	−0.30	0.194	NA	NA
Au	NA	NA	NA	NA
Hg	0.23	0.338	−0.40	0.508
Tl	−0.42	0.064	0.14	0.823
Pb	−0.13	0.593	−0.80	0.108
Bi	−0.11	0.657	0.64	0.250
Th	**−0.52**	**0.020**	NA	NA
U	0.04	0.885	0.74	0.156

Abbreviations: MMSE, Mini-Mental Status Examination; aMCI, amnestic mild cognitive impairment; AD, Alzheimer’s disease; NA, not applicable; # Adjusted for age, education level and body mass index; * defined as ([the second assessment − the first assessment]/follow up year).

## Data Availability

The datasets obtained and/or analyzed during the current study are available from the corresponding author upon reasonable request.

## Supplementary Materials

The following supporting information can be downloaded at: https://www.mdpi.com/article/10.3390/jcm11133655/s1, Table S1: The area under the curve for the trace metals on discriminating different disease groups; Table S2: The optimal cutoff of selected trace metals and the corresponding sensitivity/specificity on discriminating different disease groups.

## Abbreviations

AD: Alzheimer’s disease; aMCI: amnestic mild cognitive impairment; NIA-AA: The National Institute on Aging and Alzheimer’s Association; Aβ: β-amyloid; PET: positron emission tomography; MRI: magnetic resonance imaging; CSF: cerebrospinal fluid; GDS-S: Geriatric Depression Scale; MMSE: Mini-Mental State Examination; CDR: Clinical Dementia Rating; HVLT: Hopkins Verbal Learning Test; HIS: Hachinski Ischemia Scale; ICP-MS: inductively coupled plasma–mass spectrometry; t-Tau: total Tau protein; p-Tau: phosphorylated Tau protein (Serine 181); α-Syn: α-synuclein and IMR: immunomagnetic reduction.
