# Supplementary material for: A Metallomic Approach to Assess Associations of Plasma Metal Levels with Amnestic Mild Cognitive Impairment and Alzheimer’s Disease: An Exploratory Study
Source: J Clin Med. 2022 Jun 24;11(13):3655. doi: 10.3390/jcm11133655 (PMC9267221; doi:10.3390/jcm11133655)
Supplement: Supplementary file 1 [file jcm-11-03655-s001.zip › jcm-1761681-supplementary.pdf]

Supplemental Table S1. The area under the curve for the trace metals on discriminating different disease groups

	Area under the curve (95% CI)		
Trace metal	aMCI vs. Control	AD vs. Control	AD vs. aMCI
Li	70.1 (48.2–92.0)	63.9 (34.5–93.2)	57.1 (29.3–84.8)
Be	49.0 (25.3–72.8)	55.6 (25.7–85.4)	54.9 (30.2–79.6)
B	**97.6 (93.2–100)***	**100 (100–100)***	**69.6 (50.5–88.6)***
Al	**70.1 (51.5–88.6)***	66.7 (37.0–96.3)	57.9 (35.7–80.0)
Ca	**79.2 (62.6–95.9)***	**80.6 (58.1–100)***	50.5 (26.8–74.3)
V	68.8 (48.4–89.2)	66.7 (38.6–94.8)	57.3 (36.2–78.5)
Cr	58.5 (38.1–78.8)	48.6 (16.4–80.8)	42.4 (14.7–70.1)
Mn	63.8 (44.3–83.3)	**84.7 (60.1–100)***	**80.2 (61.5–98.8)***
Fe	69.6 (47.4–91.7)	69.4 (38.8–100)	49.5 (24.1–74.8)
Co	69.1 (47.1–91.1)	**86.1 (66.4–100)***	**72.3 (53.9–90.6)***
Ni	65.2 (43.1–87.3)	54.2 (24.5–83.9)	69.0 (45.9–92.1)
Cu	**74.4 (53.9–94.9)***	54.2 (24.0–84.4)	**72.8 (51.0–94.7)***
Zn	56.0 (28.6–83.4)	54.2 (24.5–83.8)	52.7 (26.7–78.7)
Ga	66.7 (45.7–87.6)	**88.2 (73.0–100)***	62.8 (42.4–83.1)
Ge	54.1 (34.7–73.6)	**66.7 (50.3–83.0)***	**69.6 (59.4–79.8)***
As	58.5 (32.5–84.4)	45.8 (15.9–75.7)	57.9 (34.0–81.8)
Se	58.7 (36.6–80.8)	72.2 (46.1–98.4)	**81.5 (63.1–99.9)***
Rb	54.1 (31.8–76.4)	59.7 (27.4–92.0)	56.5 (26.6–86.4)
Sr	61.8 (40.1–83.6)	70.8 (43.7–97.9)	53.3 (32.0–74.5)
Zr	**96.6 (91.2–100)***	**95.8 (87.7–100)***	56.0 (30.9–81.1)
Mo	64.5 (43.4–85.6)	51.4 (20.3–82.5)	53.3 (27.3–79.2)
Ag	52.2 (25.9–78.5)	50.0 (21.5–78.5)	56.8 (30.1–83.5)
Cd	60.9 (38.0–83.8)	55.6 (25.5–85.6)	66.0 (46.1–86.0)
Sn	44.9 (25.3–64.6)	68.8 (48.2–89.3)	59.8 (45.0–74.5)
Sb	**75.4 (52.4–98.4)***	68.1 (39.5–96.6)	46.2 (20.0–72.4)
Te	60.6 (44.4–76.8)	50.0 (33.6–66.4)	62.0 (47.0–76.9)
Ba	54.1 (30.9–77.3)	71.5 (44.7–98.3)	**73.1 (54.5–91.7)***
W	**100 (100–100)***	**100 (100–100)***	52.2 (47.9–56.4)
Pt	36.0 (13.0–59.0)	56.3 (37.5–75.1)	**77.5 (64.7–90.2)***
Au	88.9 (74.5–100)	88.9 (74.5–100)	50.0 (50.0–50.0)
Hg	**80.0 (61.5–98.4)***	**94.4 (84.3–100)***	**79.9 (57.8–100)***
Tl	**73.2 (53.1–93.3)***	**81.9 (59.8–100)***	62.0 (36.6–87.3)
Pb	67.9 (46.4–89.4)	40.3 (7.7–72.8)	**77.7 (57.2–98.3)***
Bi	**76.3 (59.8–92.9)***	**92.4 (78.0–100)***	66.0 (44.8–87.3)

Trace metal	Area under the curve (95% CI)		
	aMCI vs. Control	AD vs. Control	AD vs. aMCI
Th	**96.6 (91.1–100)***	**100 (100–100)***	**67.4 (57.4–77.3)***
U	**72.2 (54.1–90.4)***	**84.7 (65.4–100)***	63.0 (39.9–86.2)

Abbreviations: CI, confidence interval; aMCI, amnestic mild cognitive impairment; AD, Alzheimer's disease; * Indicated P <0.05.

Supplemental Table S2. The optimal cutoff of selected trace metals and the corresponding sensitivity/specificity on discriminating different disease groups

Contrasting disease groups / Trace metal	Cutoff	Sensitivity, % (95% CI)	Specificity, % (95% CI)
aMCI vs. Control			
B	≤73.1	91.3 (72.0 – 98.9)	100.0 (66.4 – 100.0)
Al	≤14.5	52.2 (30.6 – 73.2)	88.9 (51.8 – 99.7)
Ca	>88483	73.9 (51.6 – 89.8)	77.8 (40.0 – 97.2)
Cu	>843	78.3 (56.3 – 92.5)	66.7 (29.9 – 92.5)
Zr	≤0.71	82.6 (61.2 – 95.0)	100.0 (66.4 – 100.0)
Sb	≤7.80	87.0 (66.4 – 97.2)	66.7 (29.9 – 92.5)
W	≤4.29	100.0 (85.2 – 100.0)	100.0 (66.4 – 100.0)
Hg	≤2.63	87.0 (66.4 – 97.2)	66.7 (29.9 – 92.5)
Tl	≤0.05	87.0 (66.4 – 97.2)	55.6 (21.2 – 86.3)
Bi	≤0.03	52.2 (30.6 – 73.2)	100.0 (66.4 – 100.0)
Th	≤1.12	91.3 (72.0 – 98.9)	100.0 (66.4 – 100.0)
U	≤0.01	56.5 (34.5 – 76.8)	88.9 (51.8 – 99.7)
AD vs. Control			
B	≤47.1	100.0 (63.1 – 100.0)	100.0 (66.4 – 100.0)
Ca	>87064	87.5 (47.3 – 99.7)	66.7 (29.9 – 92.5)
Mn	≤0.71	87.5 (47.3 – 99.7)	88.9 (51.8 – 99.7)
Co	≤0.17	100.0 (63.1 – 100.0)	77.8 (40.0 – 97.2)
Ga	≤0.04	87.5 (47.3 – 99.7)	77.8 (40.0 – 97.2)
Ge	≤0.00	100.0 (63.1 – 100.0)	33.3 (7.5 – 70.1)
Zr	≤1.29	100.0 (63.1 – 100.0)	77.8 (40.0 – 97.2)
W	≤0.00	100.0 (63.1 – 100.0)	100.0 (66.4 – 0.0)
Hg	≤1.42	87.5 (47.3 – 99.7)	88.9 (51.8 – 99.7)
Tl	≤0.01	62.5 (24.5 – 91.5)	100.0 (66.4 – 100.0)
Bi	≤0.03	87.5 (47.3 – 99.7)	88.9 (51.8 – 99.7)
Th	≤0.00	100.0 (63.1 – 100.0)	100.0 (66.4 – 100.0)

Contrasting disease groups / Trace metal	Cutoff	Sensitivity, % (95% CI)	Specificity, % (95% CI)
U	≤0.01	62.5 (24.5 – 91.5)	100.0 (66.4 – 100.0)
AD vs. aMCI			
B	≤47.1	100.0 (63.1 – 100.0)	52.2 (30.6 – 73.)
Mn	≤0.71	87.5 (47.3 – 99.7)	69.6 (47.1 – 86.8)
Co	>0.17	100.0 (63.1 – 100.0)	52.2 (30.6 – 73.2)
Cu	≤797	62.5 (24.5 – 91.5)	82.6 (61.2 – 95.0)
Ge	≤0.00	100.0 (63.1 – 100.0)	39.1 (19.7 – 61.5)
Se	≤143.0	50.0 (15.7 – 84.3)	100.0 (85.2 – 100.0)
Ba	≤0.97	87.5 (47.3 – 99.7)	56.5 (34.5 – 76.8)
Pt	≤0.01	100.0 (63.1 – 100.0)	56.5 (34.5 – 76.8)
Hg	≤1.02	75.0 (34.9 – 96.8)	91.3 (72.0 – 98.9)
Pb	≤1.24	87.5 (47.3 – 99.7)	69.6 (47.1 – 86.8)
Th	≤0.00	100.0 (63.1 – 100.0)	34.8 (16.4 – 57.3)

Abbreviations: CI, confidence interval; aMCI, amnestic mild cognitive impairment; AD, Alzheimer's disease.
